# Female 3xTg-AD mice demonstrate hyperexcitability phenotype of Alzheimer’s disease in structure-function and function-behavior relationships

**DOI:** 10.1162/NETN.a.28

**Published:** 2025-10-30

**Authors:** Ziyi Wang (王子怡), Hui Li (李卉), Bowen Shi (史博文), Qikai Qin (秦琪凯), Qiong Ye (叶琼), Garth J. Thompson

**Affiliations:** iHuman Institute, ShanghaiTech University, Shanghai, China; School of Life Science and Technology, ShanghaiTech University, Shanghai, China; Hefei Institutes of Physical Science, Chinese Academy of Science, Hefei, Anhui, China; School of Pharmacy, Anhui University of Chinese Medicine, Hefei, Anhui, China

**Keywords:** Alzheimer’s disease, Brain functional-structural coupling, Brain function-behavior coupling, Potential biomarker of Alzheimer’s disease, Functional connectivity, Structural connectivity

## Abstract

Alzheimer’s disease (AD) causes cognitive decline with aging, hypothetically due to the accumulation of beta-amyloid (A*β*) plaques. The 3xTg-AD mouse model is increasingly used due to its initial absence of significant physical or behavioral impairments in youth and progressive A*β* plaque development with age. This mouse model thus provides an opportunity for comparison with human AD through two stages of study. Using wild-type (WT) and 3xTg-AD mice, aged 22 and 40 weeks (before and after the large increase in A*β* plaques), we measured functional connectivity (FC) and structural connectivity (SC) between brain regions. At 22 weeks, 3xTg-AD mice unexpectedly had higher SC and FC, and there was positive correlation between behavioral performance and FC density. By 40 weeks, SC and FC was lower in AD mice (similar to human AD patients), but the behavior-functional correlation was negative. Thus, our methods identified a shift in 3xTg-AD mice between two abnormal states relative to WT, moving from a hyperconnected to a hypoconnected state. Such a shift matches the hyperexcitability phenotype of AD observed in human patients, and thus suggests that 3xTg-AD mice can model the multistage etiology of AD of that phenotype.

## Author Summary

The phenotype of neuronal hyperexcitability in Alzheimer’s disease (AD) is well recognized. To better understand the underlying mechanisms driving this phenotype, we investigated the association between neural circuits, brain function, and behavior using the 3xTg-AD mouse model of AD. We measured memory, anxiety, functional connectivity, white matter integrity, and structural connectivity in 3xTg-AD and wild-type (WT) mice aged 22 weeks and 40 weeks, a time period over which beta-amyloid greatly accumulates. At 22 weeks, 3xTg-AD mice had stronger connections in their brains than WT mice, and these correlated with memory. At 40 weeks, 3xTg-AD mice had weaker connections than WT mice, which anticorrelated with memory. Our results are consistent with the hyperconnectivity phenotype observed in human AD patients, suggesting 3xTg-AD mice model multistage AD etiology.

## INTRODUCTION

Alzheimer’s disease (AD) is a type of dementia related to age, which substantially reduces the quality of life for millions of people worldwide, and is characterized by loss of brain volume and accumulation of beta-amyloid (A*β*) and tau protein. AD also reduces the functional connections of brain networks (Mondadori et al., 2006), and causes behavioral problems, notably anxiety in early stages and memory loss in late stages (Donovan et al., 2018; Ferretti et al., 2001). An important phenotype of AD is “hyperexcitability” where neuronal hyperactivity is observed at early stages, but progresses to hypoactivity at later stages of the disease (Mandino et al., 2022; Pistono et al., 2021; Shah et al., 2022; Targa Dias Anastacio et al., 2022).

The 3xTg-AD mouse model is increasingly used to study AD due to its initial absence of significant physical or behavioral impairments in youth, while progressively developing A*β* plaques (Oddo et al., 2003) and phosphorylated tau (Mandino et al., 2022) as it ages. The 3xTg-AD mouse model expresses three transgenes: PS1_M146V_ and APP_Swe_ related to A*β* and MAPT (tau_P301L_) related to tau (Oddo et al., 2003). APP_Swe_ and PS1_M146V_ have been linked to hyperexcitability (Busche et al., 2008; Minkeviciene et al., 2009; Palop et al., 2007), whereas MAPT has been linked to hypoexcitability (Hatch et al., 2017; Marinković et al., 2019). Therefore, the 3xTg-AD mouse model has the potential to demonstrate progression from early hyperexcitability to late hypoexcitability, which is not observed in models that only include APP_Swe_ and PS1_M146V_ (Busche et al., 2008; Targa Dias Anastacio et al., 2022). In particular, the female 3xTg-AD mouse phenotype “shows a close association with that observed in AD patients” (Belfiore et al., 2019, p. 10).

3xTg-AD mice demonstrate increased anxiety and reduced memory compared with wild-type (WT) as early as 17 weeks (Chiquita et al., 2019). Biomarkers of the structural connectivity (SC) of the brain have been observed as both higher (Falangola et al., 2022, 2025; Manno et al., 2019) and lower (Bergamino et al., 2023; Snow et al., 2017) in 3xTg-AD than WT, dependent upon the brain region and observed at ages from ∼9 to ∼90 weeks old. Lower functional connectivity (FC) in 3xTg-AD mice, as compared with WT, has been observed at early stages from ∼9 to ∼26 weeks old (Mandino et al., 2022; Manno et al., 2019). Alternate mouse models of AD that include the APP_Swe_ gene (and other amyloid precursor protein genes) show deficits in FC that emerge around ∼22 weeks and remain throughout the lifespan (Grandjean et al., 2016; Grandjean, Schroeter, He, et al., 2014) or show the AD model having a greater difference from WT at younger ages, with the deficit remaining but becoming less pronounced between ∼65 to 95 weeks (Morrissey et al., 2023); this also includes models other than 3xTg-AD that include MAPT (Mandino et al., 2025).

We hypothesize that these deficits in connectivity and behavior are affected by the sharp increase in A*β* that occurs in 3xTg-AD mice between 22 and 40 weeks (Belfiore et al., 2019; Duggan et al., 2024), possibly related to the phenotype of neuronal hyperexcitability in AD (Targa Dias Anastacio et al., 2022). Furthermore, we hypothesize that the behavioral-functional relationship, as well as the functional-structural relationship will be altered by the A*β* accumulation during this critical period.

We thus used multimodal magnetic resonance imaging (MRI) and behavioral testing to study 3xTg-AD mice at 22 and 40 weeks. We used functional MRI (fMRI) (Ogawa et al., 1990), specifically “resting state” fMRI where no overt stimuli or task is presented to measure “functional connectivity” and “functional connectivity density” (FCD) (Biswal et al., 1995; Lee et al., 2013; Smith et al., 2009; van den Heuvel & Hulshoff Pol, 2010). We used diffusion tensor imaging (DTI), to visualize the microstructure of neuron fiber in white matter (WM) and gray matter (GM) (Assaf & Pasternak, 2008; Filley & Fields, 2016; Zhang et al., 2012), including fractional anisotropy (FA) (Bergamino et al., 2023; Falangola et al., 2022, 2025; Manno et al., 2019; Snow et al., 2017). We also use DTI on ex vivo brains (allowing longer imaging time and higher spatial resolution) to trace the physical links between brain regions, “structural connectivity” (Dutta & Sengupta, 2016; Zhu et al., 2014). We used two behavior tests: the open field test (OFT) to study anxiety and the novel object recognition (NOR) test to study cognition and memory (Chiquita et al., 2019). Comparisons were then made between the FC and SC, FC and FA, and FCD with behavior results in order to understand how these relationships change during the period of rapid amyloid accumulation. Our study can thus determine which models of the etiology of AD better explains how the functional-structural relationship changes as A*β* increases in the brain.

## METHODS

### AD Mouse Model

The mice were purchased from Beijing Vitalstar Biotechnology Co., Ltd. The 3xTg-AD mice were maintained on the C57BL6/129SvJ hybrid background. The study employed female 3xTg-AD mice, which have more A*β* plaques and tau protein accumulation than males (Carroll et al., 2010; Pairojana et al., 2021) and age-matched control female C57BL/6N mice, aged 22 weeks and 40 weeks, with 10 mice per group (AD_22_, WT_22_, AD_40_, WT_40_). Only two time points were used to maximize statistical power and apply the reduction principle (Hubrecht & Carter, 2019); these specific time points were chosen based on previous work that indicates that they are sufficient to examine the state prior to, and succeeding, the large increase in A*β* plaques (Roda et al., 2020). Mice were cage-housed under a 12:12-hr light/dark cycle in a constant 25°C and 60%–70% humidity environment where mice could access food and water freely.

### Behavior Test

Mice were acclimated to the test room for 1 hr before trials and testing. OFTs were carried out when the mice were awake to test their anxiety (Kraeuter et al., 2019). Mice were put in the testing arena (70 × 70 × 70 cm) where they could move freely for 10 min. We analyzed the trace of each mouse’s movement in MATLAB R2020b (MathWorks, America). NOR tests were carried out to test the mice’s cognition and memory (Whittaker et al., 2023). Each mouse was first habituated to the testing arena (70 × 70 × 70 cm) for 10 min on two consecutive days. Next, they were allowed to explore two identical objects for 10 min. After 3 hr, they were put in the area again, with a novel object with a different shape and material in place of one of the previous objects, and they were allowed to explore the two differing objects for another 10 min. The time that the mice explored the novel object was recorded (Lueptow, 2017).

### In Vivo MRI Acquisition

The mice were anesthetized using intraperitoneal bolus injections of 25% urethane (Sigma-Aldrich, U2500) dissolved in saline, administered in two separate doses totaling 7 *μ*L/g. Multiple dosing of the bolus was used as it reduces animal mortality and dramatically improves the mouse physiological state (de Arce et al., 2023; Johnson et al., 2018; Li, Ye, et al., 2024) and thus improves FC as compared with urethane studies that used a single bolus (Grandjean, Schroeter, Batata, et al., 2014). The breath rate of mice under urethane was approximately 150–240/min, as our lab has already employed in numerous mouse studies to provide high-quality fMRI data (de Arce et al., 2023; Johnson et al., 2018; Li, Fang, et al., 2024; Li, Ye, et al., 2024); however, it was significantly higher in WT_22_ than that in WT_40_ (*p* = 0.0021, two-sample *t* test) but there was no significant difference between AD_22_ and AD_40_ mice, AD_22_ and WT_22_ mice, nor AD_40_ and WT_40_ mice.

Functional MRI and diffusion tensor images were acquired on a 9.4T MRI scanner (BioSpec 94/30 USR; Bruker Biospin MRI, Ettilingen, Germany), with a four-element mouse brain surface coil and an 86-mm volume transmit coil, with ParaVision 360 V3.2. T2 Turbo RARE (Rapid Acquisition with Relaxation Enhancement) sequence was used for 2D T2-weighted brain anatomical imaging. The acquisition parameters were as follows: field of view (FOV) = 24.2 × 9.6 mm^2^, echo time/repetition time (TE/TR) = 35/2003.56 ms, number of slices = 23, slice thickness = 0.3 mm, resolution = 0.1 × 0.1 mm^2^, matrix size = 242 × 96, total scan time = 2 min 36 s.

Multiband echo-planar imaging (EPI) sequence was used to acquire T2*-weighted free induction decay images for 2D functional imaging. The acquisition parameters were as follows: FOV = 24.2 × 9.6 mm^2^, TE/TR = 13/1000 ms, matrix size = 121 × 48, number of slices = 13, slice thickness = 0.6 mm, resolution = 0.2 × 0.2 mm^2^, repetition = 480, total scan time = 8 min.

To characterize the microstructure of axon tracts, in vivo DTI was performed using 2D spin-echo EPI. The acquisition parameters were as follows: FOV = 16 × 8 mm^2^, TE/TR = 19/2500 ms, matrix size = 80 × 40, number of slices = 60, slice thickness = 0.20 mm without gap, voxel size = 0.20 × 0.20 × 0.20 mm^3^, b-value = 1,000 s/mm^2^, diffusion gradient duration/separation time (*δ*/Δ) = 3.5/10.0 ms, with a total of 32 diffusion directions and five b = 0 s/mm^2^ images acquired. Number of segments = 4, averages = 4, total scan time = 24 min 40 s.

### Preparation of Ex Vivo Samples and MRI Scan

Following the MR scan with the urethane anesthesia, mice were intracardially perfused with 5 mM Gadopentetic acid (Gd-DTPA) (Adamas 86050-77-3). The brain sample was stored in a 15-ml tube with 4% paraformaldehyde solution at 4°C overnight for fixation. After this, the sample was moved to a 15-ml tube with 5 mM Gd-DTPA saline and stored at 4°C for at least 2 weeks for rehydration. On the day of ex vivo imaging, the brain samples were transferred into a 5-ml tube filled with Galden® perfluoropolyether (Solvay Specialty Polymers). Air bubbles were removed by vacuum before scanning. Due to the long scan time under urethane anesthesia and stress of perfusion setup, two AD_22_, two WT_22_, two AD_40_, and four WT_40_ mice did not survive long enough to be perfused successfully and thus were unable to be used for ex vivo DTI.

For tractography analysis, ex vivo DTI was acquired using spin-echo EPI with the following parameters: a 3D acquisition, FOV = 12 × 7.5 × 16 mm2, TE/TR = 32.5/250 ms, matrix = 120 × 75 × 160, voxel size = 0.10 × 0.10 × 0.10 mm^3^, b = 3000 s/mm^2^, *δ*/Δ = 3.0/21.0 ms, 60 diffusion directions, 5 b = 0 s/mm^2^ images, number of segments = 4, average = 4, total scan time = 11 h 33 min 20 s. Due to animal death (see above), limitations in scanner availability, and the extremely long scan time, only five mice per group for AD_22_, WT_22_, and AD_40_, and six mice for WT_40_ were scanned using this protocol.

### fMRI Processing

For resting-state fMRI (rs-fMRI) analysis, functional images were preprocessed by brain extraction, slice-timing correction, and motion correction in MATLAB R2020b (https://uk.mathworks.com/products/matlab) with SPM12 (https://www.fil.ion.ucl.ac.uk/spm/software/spm12/). The images were adjusted as if all slices were acquired simultaneously by using slice-timing correction, and motion artifacts were eliminated by motion correction. Functional images were then aligned with anatomical images within the same subject by rigid transformation and then anatomical images were coregistered to the Allen mouse brain atlas (Lein et al., 2007) in MATLAB R2020b and BioImage Suite Web (https://bioimagesuiteweb.github.io/webapp/). This atlas provides regions of interest (ROIs) with specific Allen numbers. Due to the resolution of MRI, we combined ROIs to allow for sufficient signal. Supporting Information Table S1 indicates the brain region, abbreviation, and list of Allen numbers for ROIs combined to create each ROI used in our study. All images were smoothed with FWHM (full-width-half-maximum) of 5 to allow for better comparison between mice and to facilitate the group-based, ROI-based analysis in this study.

Group independent component analysis (group ICA) took place in MATLAB R2020b using Group ICA of the fMRI Toolbox (https://trendscenter.org/software/gift/), and the components related to cerebral spinal fluid noise were removed (Han et al., 2019).

Preprocessed data were divided into five epochs, with each epoch including 96 repetitions. We removed epochs that had a Pearson’s correlation coefficient (pcc) of <0.1 between the left and right brain regions of lower forelimb primary somatosensory cortex (S1FL). This was undertaken to improve signal quality, as the S1FL region normally has high correlation if the mouse is in a good physiological state, per our previous work (de Arce et al., 2023; Johnson et al., 2018). This resulted in the complete removal of three AD_22_, two WT_22_, one AD_40_, and two WT_40_ mice. (Note that behavioral data from these mice were not removed for calculation of average behavioral parameters.)

FC matrices were calculated using the pcc of the mean BOLD signals between different brain regions. Student’s *t* tests were calculated using MATLAB and resulting *p* values were corrected for multiple comparisons by sequential goodness of fit (SgoF) metatest for family-wise error rate (FWER) of 0.05 (Carvajal-Rodríguez et al., 2009).

Global functional connectivity density (gFCD) was calculated by creating a correlation matrix for all voxels in the brain, then counting each voxel’s number of connections with other voxels as the number of voxels it correlated with above a threshold of 0.1 as per Tomasi and Volkow (Tomasi & Volkow, 2010). Thus, gFCD reflected the level at which each voxel in the brain was an FC hub. In addition, gFCD was used to perform outlier removal. Mice whose data included greater than 3 standard deviations from whole brain mean gFCD were removed from behavior-gFCD analysis, this was only one AD_22_ mouse. (Thus, the total number removed including prior removals was four AD_22_ two WT_22_, one AD_40_, and two WT_40_ mice.)

### DTI Processing

Preprocessing: both in vivo and ex vivo DTI datasets were initially converted into 4D NIfTI images using DSI studio (January 3, 2022) as described by Yeh et al. (2019). The dataset underwent denoising using nlsam (https://nlsam.readthedocs.io/en/latest/autoapi/nlsam/index.html). Summed diffusion weighted images were utilized for brain extraction/mask delineation in BrainSuite (Version 21a) (https://brainsuite.org/). Motion and Eddy current correction procedures were conducted.

The in vivo DTI metrics, including FA as an indicator of axon integrity; mean diffusivity and axial diffusivity as an indicator of axon injury; and radial diffusivity as an indicator of myelination, were computed in DSI studio (Version 2022 Jul) using the denoised DTI dataset. Q-space diffeomorphic reconstruction (QSDR) was performed based on the Allen Mouse Brain Common Coordinate Framework Version 3 (ABA). Subsequently, the average parametric values for each 3D brain region were extracted using MATLAB scripts (Mathworks, R2021b). Prior to further analysis, mice were excluded if visual inspection of their in vivo DTI images could not clearly differentiate WM from GM, which resulted in three AD_22_, three WT_22_, one AD_40_, and three WT_40_ mice being removed from in vivo DTI results.

The ex vivo DTI connectome analysis employed QSDR reconstruction with the ABA mouse brain template. The tracking threshold was set at qa = 0.01, angular threshold at 45°, and minimum/maximum length at 1.0/40.0 mm, with terminal seeds of 1,000,000. Connectivity analysis utilized 15 ROIs derived from rs-fMRI analysis. For each mouse brain, a connectivity matrix was calculated using these 15 brain regions. Group comparison was conducted through a two-sample *t* test across ages and genotypes. To visualize the connectogram displaying significant group differences, the averaged connectivity matrix with significant differences was plotted on the website of circos (https://mkweb.bcgsc.ca/tableviewer/visualize/).

### Statistical Analysis

Statistical analysis regarding behavior and also regarding the relationship between FC and both ex vivo and in vivo DTI were calculated in GraphPad Prism (Version 8, GraphPad Software, San Diego, CA, USA). For behavior, unpaired two-tailed student’s *t* tests were used to compare the significance between different groups. The relationship between FC and both ex vivo and in vivo DTI was calculated by Pearson correlation. (Only mice whose data were neither excluded for fMRI nor ex vivo DTI were used for the FC vs. in vivo comparison, and only mice whose data were not excluded for fMRI and who survived long enough to perform brain perfusion were used for the FC vs. ex vivo comparison.) For each group, the mean slope from all linear regressions within the group was calculated. Unpaired two-tailed Student’s *t* tests were used to compare groups with the relationship between FC and in vivo DTI. Multiple comparison correction was performed with SgoF for correlation maps as described above (Carvajal-Rodríguez et al., 2009). No multiple comparison corrections were performed for the behavior test or for the relationship between FC and in vivo DTI. (Note that when matching data from the mice in the correlation between FC and in vivo DTI, five AD_22_, five WT_22_, two AD_40_, and three WT_40_ mice had data removed due to a combination of exclusion criteria described previously.)

## RESULTS

### In Vivo rs-fMRI FC

FC is an important biomarker for AD. Hyperconnectivity has been found in early-onset AD patients (Fide et al., 2022) and patients at an early stage of risk for AD (García-Colomo et al., 2024). Conversely, lowered FC has been long known in human patients diagnosed with AD (Greicius et al., 2004; Rombouts et al., 2005), which other studies have observed in 3xTg-AD mice (Mandino et al., 2022; Manno et al., 2019).

**Figure 1.**
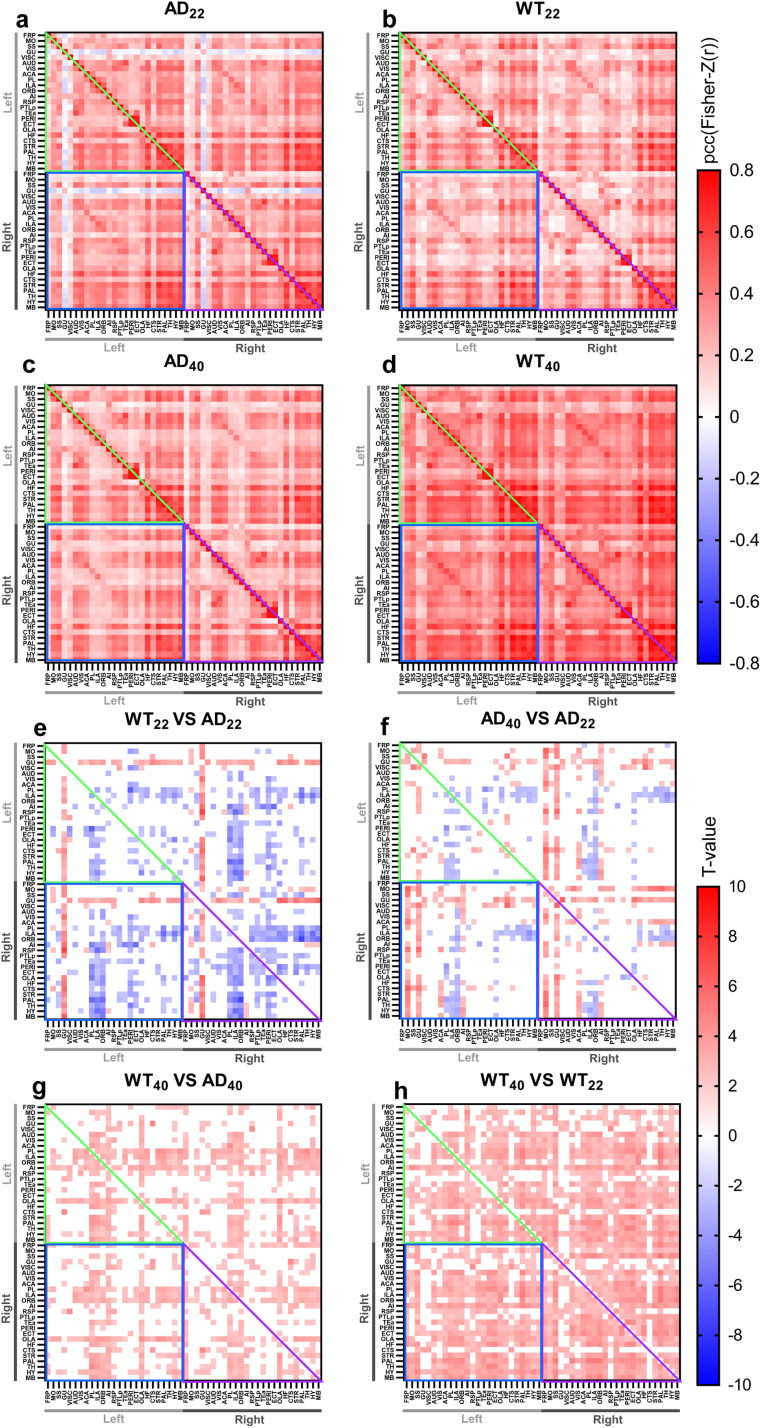
Averaged functional connectivity matrices and *t* value map comparison. (A–D) pcc is plotted for pairs of brain regions (calculated from the Fisher-Z transformation of *r* values). The light gray bar indicates left hemisphere regions; the dark gray bar highlights right hemisphere regions. The diagonal represents the connectivity of the same region, the green triangle highlights “left hemisphere connections,” the purple triangle highlights “right hemisphere connections,” and the blue square highlights “interhemispheric connections.” (E–H) *t* values from comparing WT versus AD, and 40 weeks versus 22 weeks, are plotted for pairs of brain regions. Colored blocks show the original *t* values of significant brain regions. A *t* value > 0 means the connectivity of the former group (group names shown above the plot) is higher than the latter group and vice versa. White blocks indicate not statistically significant. (*t* test, *p* < 0.05, FWER corrected by SgoF, only correlations passing SgoF are shown in color.) The ROI names are shown in Supporting Information Table S1. AD_22_: *N* = 7; WT_22_: *N* = 8; AD_40_: *N* = 9; WT_40_: *N* = 8.

For brain function, a comparison of the FC maps between AD and WT mice at 22 and 40 weeks revealed a number of important observations. For all tested regions, in AD_22_ and AD_40_ mice, the gustatory areas (GU) showed a negative correlation with other regions, but in WT_22_ and WT_40_ mice, the GU showed a positive correlation with all tested regions (Figure 1A–D). Next, we compared the changes of FC between different groups. First, we found the FC of the frontal pole (FRP), somatomotor areas (MO), and GU with other regions were significantly lower in AD_22_ mice compared with WT_22_ mice, while the FC of the other cortical regions and other subcortical regions with other brain regions were significantly higher in AD_22_ mice (Figure 1E). The FC between most pairs of brain regions were significantly lower in AD_40_ mice than in WT_40_ mice (Figure 1F). While FC of the MO and GU with other regions was found to be lower in AD_22_ mice than AD_40_ mice, the FC of the infralimbic area (ILA), orbital area (ORB), and olfactory area (OLA) with other regions were significantly higher in AD_22_ mice (Figure 1G). In WT mice, the FC was significantly lower at 22-weeks (Figure 1H).

These results revealed that in normal aging, FC increased from 22 to 40-weeks, but with AD this increase only existed in MO and GU regions. In addition, compared with WT mice, the FC was higher in AD at the younger age.

### Ex Vivo DTI SC

To test SC that was comparable to our FC results, brain regions were chosen for ex vivo DTI SC based on their significance in the in vivo rs-fMRI FC results. Thus, the brain regions chosen were the agranular insular area (AI), ectorhinal area, FRP, GU, ILA, ORB, perirhinal area, temporal association area (TEa), and visceral area cortical regions and the amygdala (AMY), hippocampus formation, hypothalamus (HY), OLA, pallidum (PAL), and thalamus (TH) subcortical regions.

**Figure 2.**
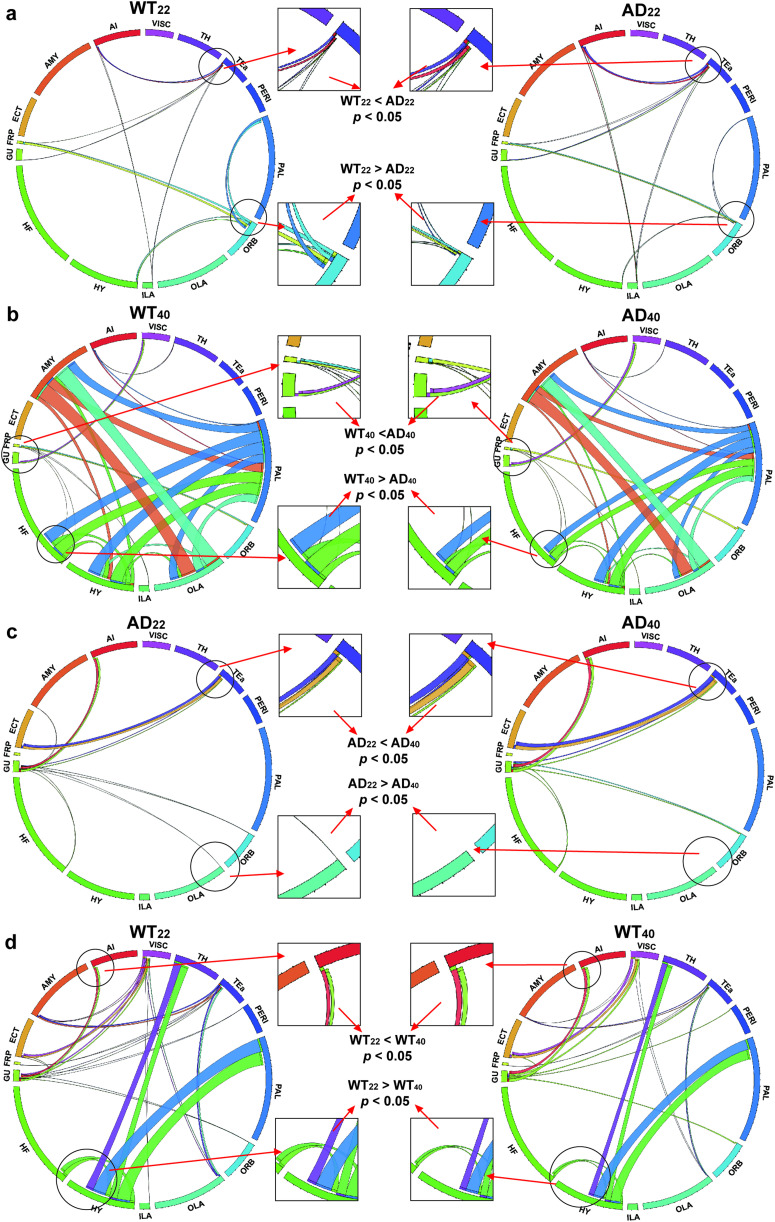
Brain structural connectivity from ex vivo DTI. (A–D) The left and right columns illustrate a part of the average connectivity map for the group as labeled. Line thickness represents the SC strength for the group as labeled, relative to the other group on the row, for example, if the line is thicker on the left column than the right, then the left column has a higher SC. Different colors of the lines and perimeter indicate different brain regions as labeled around the perimeter. Lines are drawn between brain regions only where there is a significant (*p* < 0.05) difference between the groups in the left and right columns. Selected comparisons are zoomed in to illustrate which group had significantly greater SC. Only ROIs that were selected due to significant differences in FC were tested (see the main text). Brain region names are shown in Supporting Information Table S2. AD_22_: *N* = 5; WT_22_: *N* = 5; AD_40_: *N* = 5; WT_40_: *N* = 6.

We calculated the SC between our chosen regions and compared the results. We found that the SC between ORB with HY and PAL in AD_22_ mice were significantly lower compared with WT_22_ mice, while the SC between FRP, GU, TEa, AI, and ILA were significantly higher (Figure 2A). The SC between cortical regions of AD_40_ mice were significantly lower than in WT_40_ mice, whereas the SC between subcortical regions were significantly higher (Figure 2B). The SC was found to be significantly lower in AD_22_ than AD_40_ mice while SC between GU and AMY was only detectable in AD_22_ mice, not AD_40_ mice (Figure 2C). In WT mice, the SC of the TEa, TH, HY, and PAL between other regions were significantly higher for WT_22_, while the SC of the cortical regions with other regions were significantly lower (Figure 2D). From these results, we concluded that the SC increases in AD mice, but otherwise, the SC between cortical regions increases with age and SC between subcortical regions decreases with age in normal mice.

AD_22_ mice had lower SC in subcortical regions but higher SC in cortical regions than WT_22_ mice, while conversely, AD_40_ mice had higher SC in subcortical regions and lower SC in cortical regions than WT_40_ mice (Figures 2A and 2B). These results suggest that the SC increases as the FC declines due to the effects of A*β*-based injury on brain function.

### Relationship Between FC and SC

**Figure 3.**
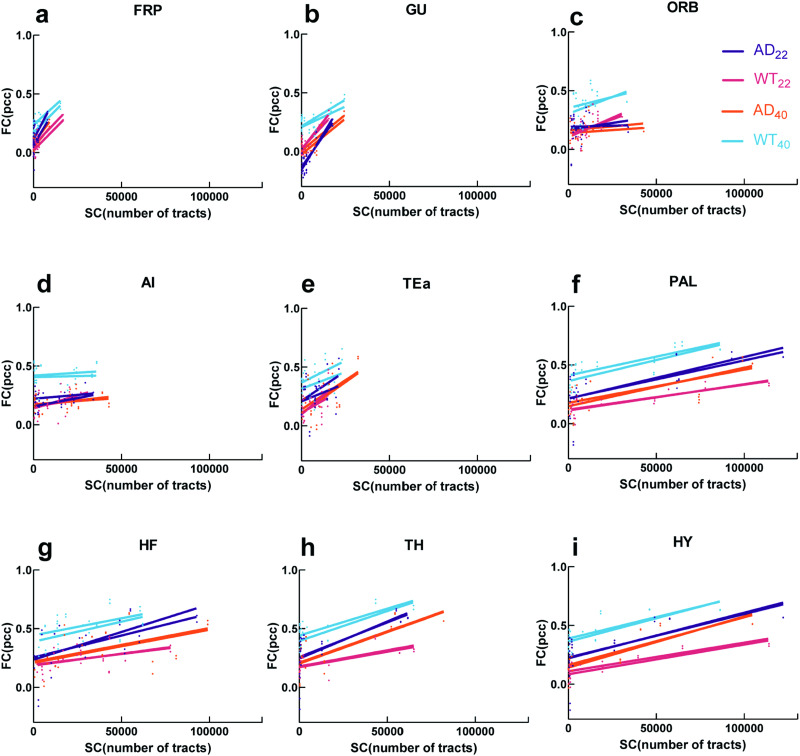
Correlation between FC (pcc) and SC (number of tracts that pass two ROIs). (A–I) Relationship between FC and SC of the labeled regions and other regions. Lines of the same color represent the left and right hemispheres of the brain. Each dot represents the mean FC and SC of two matched bilateral regions of each group. For example, the correlation in FRP is the SC between FRP and other regions and FC between FRP and other regions from both left and right hemispheres. The *r* and *p* values are shown in Supporting Information Table S2.

Functional-structural coupling has great potential for predicting AD (Hari et al., 2023; Qian et al., 2015), and previous studies showed the FC-SC had a coupling change between mild cognitive impairment and AD patients (Qian et al., 2015). Therefore, we compared the relationship between in vivo rs-fMRI-based FC and ex vivo DTI-based SC using nine brain regions for which they showed connectivity significance in both FC and SC. We found that FC increased as SC increased in all nine regions (Figure 3).

The correlation in FRP was weak in AD_22_ mice, but higher in AD_40_ mice. The FC-SC correlation in GU was weak in WT_40_ mice. The FC-SC correlation in PAL was weak in AD mice as compared with WT mice at 22 weeks and 40 weeks. The correlation in TH was weak at 22 weeks versus 40 weeks. The correlations in HY were weak in AD_22_ and WT_40_ mice (Supporting Information Table S2). Overall, these results showed correlated FC and SC within cortical regions, and also that higher SC supported higher FC.

### Relationship Between FC and Microstructure

The most frequently used parameter calculated from DTI is FA, which is demonstrated to be altered in 3xTg-AD mice (Bergamino et al., 2023; Falangola et al., 2022, 2025; Manno et al., 2019; Snow et al., 2017). Therefore, we chose to compare FA to FC within the brain regions previously chosen.

**Figure 4.**
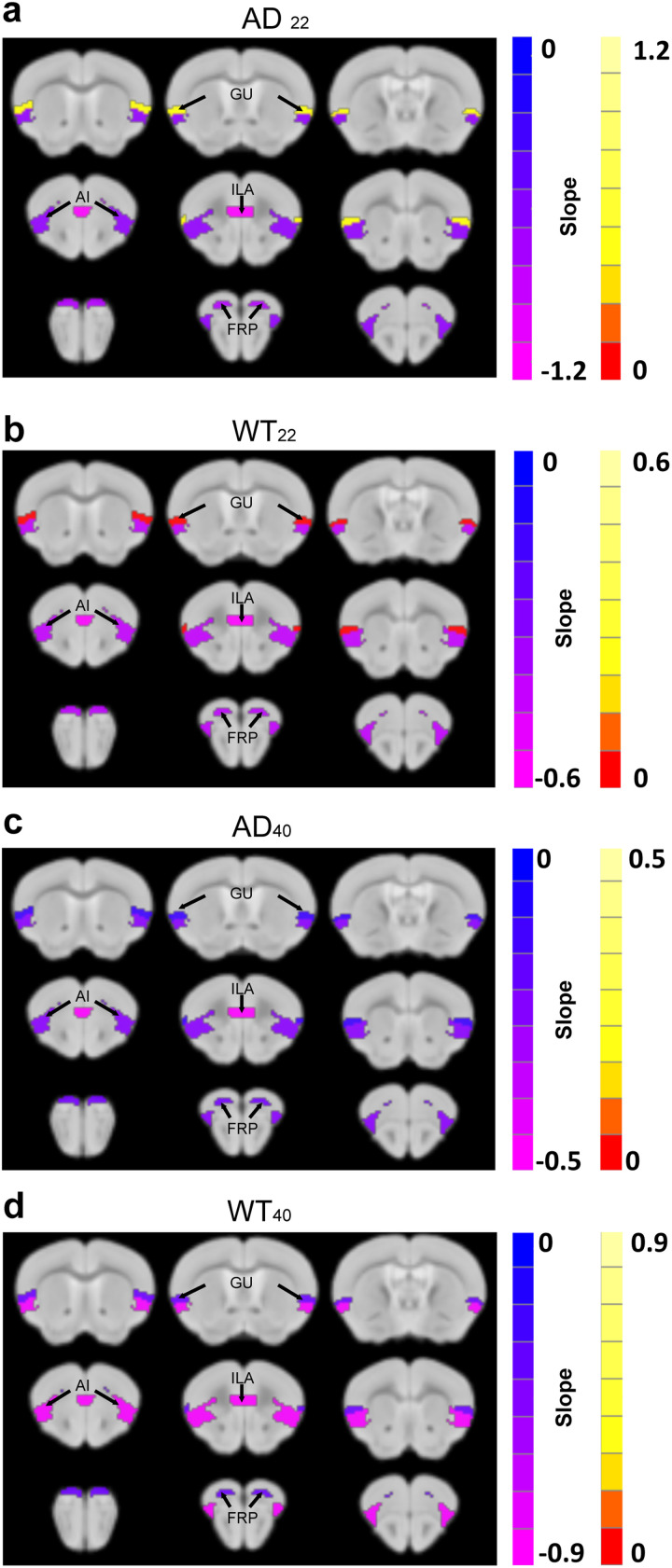
Linear regression results between FC and FA. (A–D) The slope of the linear regression between FC and in vivo FA from four brain regions as labeled and indicated with arrows. Color indicates the slope of the linear regression (see also Supporting Information Table S3). AD_22_: *N* = 5; WT_22_: *N* = 5; AD_40_: *N* = 8; WT_40_: *N* = 7.

We used in vivo DTI to measure the microstructure of the brain and to study the relationship between FC and microstructure of brain parenchyma. FA as the parameter of in vivo DTI was used to measure the integrity of WM. The FRP, GU, ILA, and AI cortical regions were chosen to study the relationship between FC and the microstructure of brain parenchyma because these regions showed both FC and SC significance in prior analyses (Figures 1E and 1F, Figure 2A–D). The GU region showed a positive correlation between FC and FA in both AD_22_ and WT_22_ mice (Figures 4A and 4B), but a negative correlation at 40-weeks (Figures 4C and 4D). The FRP, ILA, and AI regions showed a negative correlation (Figure 4, Supporting Information Table S3) where an FC increase accompanied an FA decrease.

### Behavior and Brain Function

**Figure 5.**
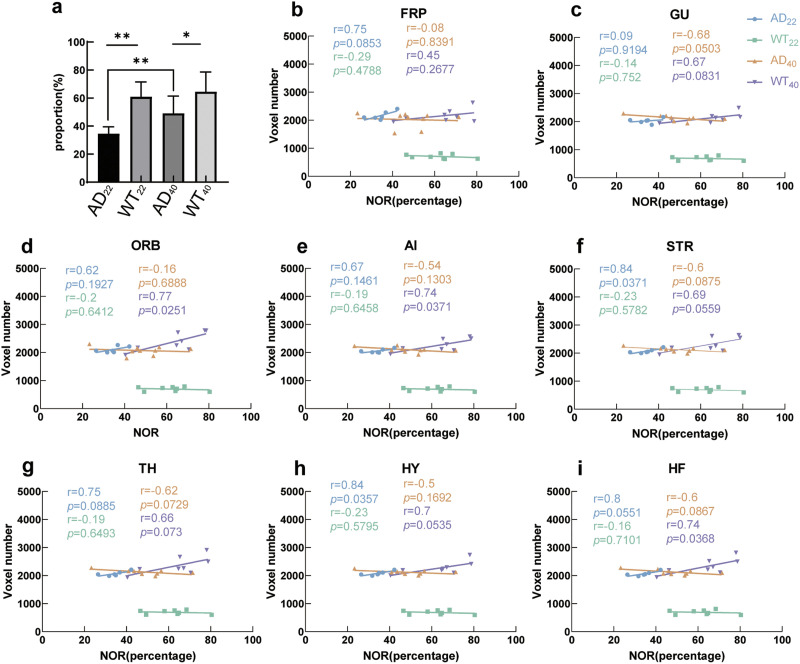
Linear regression between behavior (NOR) and brain connectivity (gFCD). (A) Bar graph showing the proportion of time spent exploring the novel object, with mean and standard error shown. (B–I) Correlation between NOR and gFCD of the region shown. The *y*-axes are gFCD of the brain regions in terms of number of voxels. Each dot is one subject of each group of the tests; the line of the same color is the linear regression of one group. The *r* and *p* values (the coefficient of determination) are shown in the same color assigned to the group. AD_22_: *N* = 6; WT_22_: *N* = 8; AD_40_: *N* = 9; WT_40_: *N* = 8.

We used the NOR test to study mouse memory and the relationship between memory and brain function. Here, the greater the proportion of time a mouse explored the novel object, the better their memory. Using this approach, we found that AD_22_ mice had significantly worse memory during the task compared with AD_40_ mice (*p* = 0.003). Comparing AD and WT mice at both time periods, AD mice showed significantly worse memory than WT mice (*p* < 0.0001, *p* = 0.0180) (Figure 5A). AD_22_ and WT_40_ mice groups showed a positive correlation between NOR and gFCD, while the WT_22_ and AD_40_ mice had a negative correlation (Figure 5B–I). These results revealed a correlation between better memory and more brain connectivity in the AD_22_ and WT_40_ mice, whereas in AD_40_ mice, better memory was correlated with less brain connectivity.

Interestingly, while the slope of the linear relationship between FC and memory went from positive to negative during aging in WT mice, similar to results from human subjects (King et al., 2018), this modality was opposite for AD mice at both stages, going from negative to positive.

**Figure 6.**
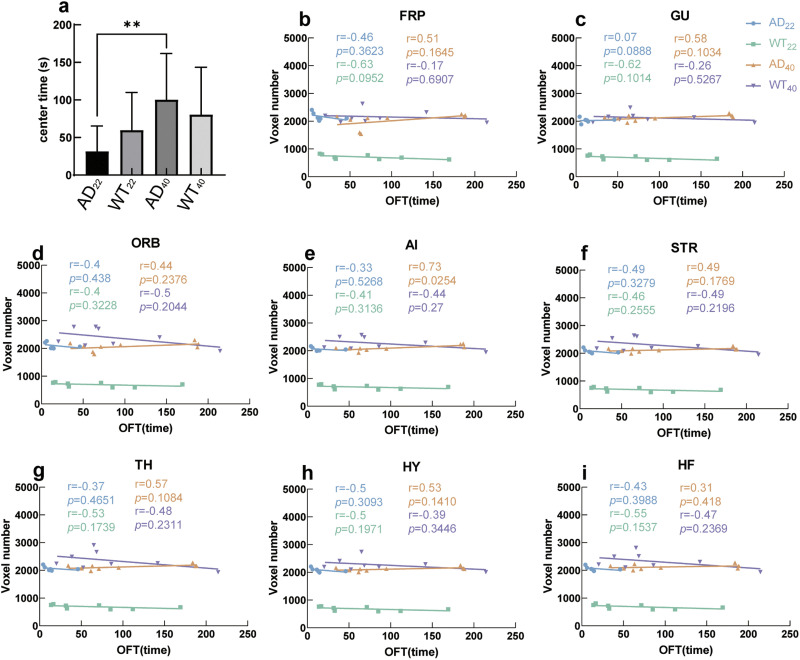
Linear regression between anxiety (OFT) and brain connectivity (gFCD). (A). Bar graph showing the proportion of time spent in the center of the field with mean and standard error shown. (B–I) Correlation between OFT and gFCD of the region shown. Each dot is one subject of each group of the tests; the line of the same color is the linear regression of one group. The *r* and *p* (the coefficient of determination) is shown in the same color assigned to the group. AD_22_: *N* = 6; WT_22_: *N* = 8; AD_40_: *N* = 9; WT_40_: *N* = 8.

We used the OFT to study the anxiety level of mice and the relationship between anxiety and brain function. Here, the longer the proportion of time the mouse stayed in the center of the arena, the less anxiety the mouse showed. Using this method, we found that AD_22_ mice had significantly more anxiety during the task than AD_40_ mice (*p* = 0.0046) (Figure 6A). The AD_22_ and WT_40_ mice showed a negative correlation between OFT and gFCD, while WT_22_ and AD_40_ mice had a positive correlation between OFT and gFCD (Figure 6B–I). Our results reveal that more anxiety is correlated with more brain connectivity in AD_22_ and WT_40_ mice, whereas in AD_40_ mice, more anxiety correlated with less brain connectivity.

We observed gFCD in WT_22_ mice was lower on average than in WT_40_ mice (Figures 5 and 6). This may be because gFCD correlates highly with glucose and oxygen metabolism (Thompson et al., 2016; Tomasi et al., 2013; Wang et al., 2024; Xu et al., 2022), and, in WT mice, metabolism dramatically increases from 22 to 40 weeks, but in AD mouse models, this increase was not observed (Kuhla et al., 2020; Waldron et al., 2017).

## DISCUSSION

### Functional Deficit Compensation and Neuronal Hyperexcitability

Our results are consistent with the hypothesis that the increase in FC of subcortical regions is caused by a compensatory effect at the early stage due to the slight accumulation of A*β* plaques and tau protein aggregation (Aganj et al., 2020; Gaubert et al., 2019; Mormino et al., 2011). In a previous study using a mouse model of AD that included APP_Swe_ and another amyloid precursor protein, the authors found enhanced neuronal hyperactivity in the AD mice related to intracellular Ca^2+^ storage (Lerdkrai et al., 2018). In the context of our study’s results, this could indicate a compensatory effect in the subcortex that results in the cortex being more vulnerable than the subcortical regions.

Furthermore, our FC results and functional-behavioral correlation results suggest that the 3xTg-AD mouse model demonstrates the hyperexcitability phenotype of AD. This is likely because A*β*-related genes shown to increase excitability in neocortical (Minkeviciene et al., 2009) and hippocampal (Palop et al., 2007) cells, whereas tau-related genes shown to decrease excitability in neocortical (Marinković et al., 2019) and hippocampal (Hatch et al., 2017). Our results also support that the degree of A*β* accumulation may shift the conditions from the hyperexcitable to hypoexcitable stage. On a cellular level, neurons that are proximal to high A*β* concentrations will demonstrate hyperexcitability (Busche et al., 2008), and the resulting hyperactivity can increase A*β* accumulation (Cirrito et al., 2005), thus creating a “vicious cycle” (Targa Dias Anastacio et al., 2022, p. 7) that can lead to cell death.

Regarding aging, in WT mice our results replicate many previous studies that show an increase in FC over the 22- to 40-week period (Egimendia et al., 2019; Mandino et al., 2025; Morrissey et al., 2023; Vasilkovska et al., 2023). Similarly, studies of alternate AD mouse models have shown FC in those AD mouse models also not having increased by 40 weeks (while later increases do occur, the level of FC in AD mice never again reaches the level of WT mice) (Mandino et al., 2025; Morrissey et al., 2023). The primary difference regarding aging in our results is the observation of hyperconnectivity at 22 weeks.

Conversely, hyperconnectivity may be a symptom rather than a compensatory mechanism. Younger patients with AD are actually at higher risk of developing seizures and myoclonus (Vossel et al., 2017), suggesting that hyperconnectivity might be a pathology itself.

### The Relationship Between FC and SC

Our results demonstrated that, for most brain regions and groups, FC has a positive correlation with SC. Previous studies have also shown that SC increases are usually correlated with FC increases in human subjects (Honey et al., 2009; Koch et al., 2002; Li et al., 2012), and our results support their conclusions. In cases where there is a weak correlation between FC and SC, this result may indicate that there exists indirect SC of these two regions, that they are correlated functionally but not connected directly structurally, rather both connect to some third region. In these cases, where the brain FC is not generated via a direct structural connection, the regions with greater correlation between FC and SC are at greater risk to be more severely injured in disease (Deco et al., 2014; Straathof et al., 2020).

Considering the cases of negative FA-FC correlation, the A*β* plaques and tau protein aggregation in the 3xTg-AD and other mouse models can cause neuroinflammation near microglia, and such neuroinflammation can cause synapse loss (Nakamura et al., 2021; Rajendran & Paolicelli, 2018). Considering the cases of positive FA-FC correlation, in human research, FA can increase with age due to the ensheathment of oligodendrocytes around axons (Dubois et al., 2008; Hüppi & Dubois, 2006; Qiu et al., 2015); however, the FC may not increase in cases where the connection pathways have been damaged (Cho et al., 2009; Terry et al., 1991).

### Memory Loss in 3xTg-AD Model

Surprisingly, we observed that AD_22_ mice had worse memory than AD_40_ mice (Figure 5A). We consider two possibilities for this. First, performance on memory tasks by mice is highly influenced by their level of anxiety (Li et al., 2023; Vijaya et al., 2024). In our study, AD_22_ mice had greater anxiety than any other group, and AD_40_ mice had lower anxiety than any other group (Figure 6A).

Second, it is possible that 40 weeks is not long enough to fully measure aging-related memory loss in the 3xTg-AD model. We observed worse memory in AD mice at both 22 weeks and 40 weeks relative to WT. This is supported by Chiquita et al. (2019), whose results suggest that memory deficits occur across the lifespan, but aging-related memory loss in 3xTg-AD mice has not occurred even by 69 weeks.

### Data Quality Considerations

Certain groups have argued that rather than only excluding data between epochs of low bilateral correlation (as we have done here), that data should also be excluded as “not biologically plausible” if there is high correlation between sensory (e.g., primary somatosensory cortex barrel field [Sibf]) and associative cortex (e.g., anterior cingulate area [ACA]) (Grandjean et al., 2020, 2023). We disagree with this interpretation because there is strong evidence from human studies that periods of high correlation between sensory cortex and associative cortex is a natural part of inherent shifts in attention (Thompson et al., 2013; Watters et al., 2025), which also occur under anesthesia (Thompson et al., 2014) particularly within the short epochs we used for data quality measurement (96 repetitions) (Murakami et al., 2005; Neville & Haberly, 2003).

However, in the interest of allowing comparison to studies from these groups, we have also created a “quality control” graph shown in Supporting Information Figure S1, and repeated the creation of Figure 1, except with the additional exclusion of data which has high correlation between Sibf and ACA, shown in Supporting Information Figure S2.

### Gustatory Cortex as Potential Early Biomarker for Human AD

We found that the FC of the cortical regions especially GU of AD_22_ (∼5 months old) mice had already decreased and that SC had already increased, suggesting that these early correlated changes may be an important biomarker of disease. The decline in FC of the GU region suggests that the AD mice have loss of gustatory function. The relationship between FC and SC in GU was a positive linear relationship in 22-week-old mice, but a negative linear relationship in 40-week-old mice. The WT_40_ mice had a higher negative slope than AD_40_ mice while the WT_22_ mice showed a lower positive slope than AD_22_ mice (Figure 4, Supporting Information Table S3). Thus, the gustatory loss is also worth investigating in human AD.

### Limitations

Due to our specific focus, our study had several limitations. First, we only included two time points, but our results and comparison to alternate AD mouse models (Mandino et al., 2025; Morrissey et al., 2023) suggest examining a later time point where 3xTg-AD mouse FC has increased may also be interesting. We used preserved brains for long-term DTI rather than histology, so we lack direct measurements of A*β* and tau histology, which could be correlated on an individual-mouse level with behavior and so forth (Chiquita et al., 2019). Urethane anesthesia is a nonsurvival protocol, thus, longitudinal work would require another anesthesia. Urethane may produce FC that differs from other anesthesia (e.g., compare Supporting Information Figure S1 with Grandjean et al., 2020, Figure 5D). However, future work is needed to compare the multiple bolus urethane protocol (de Arce et al., 2023; Johnson et al., 2018) to other protocols.

In addition to A*β* and tau, the 3xTg-AD model includes an extensive pathology, including gut inflammation (Chen et al., 2021), overly activated hypothalamic-pituitary-adrenal stress axis (Hebda-Bauer et al., 2013), and altered glucose, glutamate, and taurine metabolism (Chiquita et al., 2019; Pfitzer et al., 2025; Sancheti et al., 2014). These factors may also hypothetically affect observed changes in FC and future work that combines treatment of AD in the 3xTg-AD mouse model with functional imaging can potentially separate such effects by selectively relieving them (e.g., excision of the vagus nerve relieves gut symptoms [Chen et al., 2021]).

Our study only used female 3xTg-AD mice because they exhibit significantly greater A*β* burden and altered behavior than male 3xTg-AD mice (Carroll et al., 2010), and also because they exhibit more reliably similar symptoms to human AD patients (Belfiore et al., 2019). However, this may indicate that aging in these mice more strongly resembles AD onset in women than in men. For example, only female carriers of the apolipoprotein E (APOE) ε4 gene demonstrated a significantly higher MRI “hyperexcitation indicator,” whereas results for male carriers were nonsignificant (Fortel et al., 2020). Conversely, increased FC has also been observed in studies of first-degree relatives of AD patients with no effect due to sex or APOE ε4 (Ramírez-Toraño et al., 2021). Future study of male 3xTg-AD mice may be needed to better study AD progression in male patients.

### Conclusion

In conclusion, through examining the changes to the structural-functional and functional-behavioral relationships between two ages in 3xTg-AD mice, we have demonstrated that, rather than degrading from a normal state to an abnormal state, they shift from one abnormal state to another, with the early state indicating hyperexcitability and the latter state better corresponding to diagnosed human patients with AD. Our results thus support models of the etiology of AD that include multiple stages, particularly those in which earlier stages have the AD versus healthy results reversed (Demetrius & Simon, 2012; Rubinski et al., 2020). Specifically, the 3xTg-AD mouse model thus is well-suited to studying the hyperexcitability phenotype of AD (Targa Dias Anastacio et al., 2022).

## ACKNOWLEDGMENTS

Dr. Qikai Qin is currently affiliated with Athinoula A. Martinos Center for Biomedical Imaging, Massachusetts General Hospital, and Department of Radiology, Massachusetts General Hospital and Harvard Medical School.

The authors would also like to thank Prof. Kurt Wüthrich for his mentoring and the lab of Prof. Ji Hu from ShanghaiTech University for providing resources and training for mouse behavior measurement and analysis.

## SUPPORTING INFORMATION

Supporting information for this article is available at https://doi.org/10.1162/NETN.a.28.

## AUTHOR CONTRIBUTIONS

Ziyi Wang: Data curation; Formal analysis; Investigation; Methodology; Supervision; Writing – original draft. Hui Li: Conceptualization; Funding acquisition; Methodology. Bowen Shi: Methodology. Qikai Qin: Methodology. Qiong Ye: Conceptualization; Formal analysis; Funding acquisition; Investigation; Methodology; Resources; Writing – original draft; Writing – review & editing. Garth J. Thompson: Conceptualization; Funding acquisition; Investigation; Methodology; Resources; Supervision; Writing – review & editing.

## FUNDING INFORMATION

Hui Li, National Natural Science Foundation of China (https://dx.doi.org/10.13039/501100001809), Award ID: No. 3210055. Garth J. Thompson, National Natural Science Foundation of China (https://dx.doi.org/10.13039/501100001809), Award ID: No. 81950410637. Qiong Ye, Collaborative Key Foundation of Hefei Science Center, Award ID: No. 2022HSC-CIP003. Garth J. Thompson, ShanghaiTech University, the Shanghai Municipal Government.

## ETHICS DECLARATION

All procedures were approved by the Institutional Animal Care and Use Committee at Hefei Institutes of Physical Science, Chinese Academy of Sciences.

## DATA AVAILABILITY

Data and code will be made available upon reasonable request, please contact contact@garththompson.com and qiong.ye@hmfl.ac.cn.

## Supplementary Material



## TECHNICAL TERMS

3xTg-AD mouse model: A mouse model used to study Alzheimer’s disease, they express three human transgenes: PS1_M146V_, APP_Swe_, and MAPT.
Structural connectivity (SC): The white matter tracts that connect brain regions, physical features of the brain.
Functional connectivity (FC): The degree to which the functional signals between two or more brain regions are synchronized in time.
Functional magnetic resonance imaging (fMRI): An MRI method that uses the ratio of deoxyhemoglobin versus hemoglobin to measure brain function.
Diffusion tensor imaging (DTI): An MRI method that measures the anisotropic diffusion of water to characterize tissue microstructure and track white matter fibers.
Fractional anisotropy (FA): The degree to which white matter within a voxel is anisotropic (aligned in a particular direction).
Open field test (OFT): A rodent behavioral test whereby more time spent in the open area indicates less anxiety.
Novel object recognition (NOR): A rodent behavioral test whereby more time investigating a novel object indicates better memory.
Global functional connectivity density (gFCD): For one voxel, the number of other voxels with which it has high functional connectivity.
Hyperexcitability phenotype: When, in Alzheimer’s disease, neural activity or connectivity is high at early stages, or for early onset.
