# Supplementary material for: Female 3xTg-AD mice demonstrate hyperexcitability phenotype of Alzheimer’s disease in structure-function and function-behavior relationships
Source: Netw Neurosci. 2025 Oct 30;9(4):1199–220. doi: 10.1162/NETN.a.28 (PMC12594488; doi:10.1162/NETN.a.28)
Supplement: Supplementary file 1 [file netn-9-4-1199-s001.pdf]

Supplemental data for:

Female 3xTg-AD mice demonstrate hyperexitability phenotype of Alzheimer's disease in structure-function and function-behavior relationships

Abbreviated Title: 3xTg-AD mice and hyperexcitability phenotype

Ziyi Wang (王子怡)[1, 2], Hui Li (李卉)[1], Bowen Shi (史博文)[1, 2], Qikai Qin (秦琪凯)[1, 2], Qiong Ye (叶琼)[3] and Garth J. Thompson[4]

1. iHuman Institute, ShanghaiTech University, Shanghai, China 201210

2. School of Life Science and Technology, ShanghaiTech University, Shanghai, China 201210

3. Hefei Institutes of Physical Science, Chinese Academy of Science, Hefei, Anhui, China 230031

4. School of Pharmacy, Anhui University of Chinese Medicine, Hefei 230038, China

Correspondence should be addressed to Qiong Ye at qiong.ye@hmfl.ac.cn or Garth J. Thompson at contact@garththompson.com.

Table of contents:

Supplementary Figures 2
  Figure S1. 2
  Figure S2. 4
Supplementary Tables 5
  Table S1. 5
  Table S2. 6
  Table S3. 7

Supplementary Figures

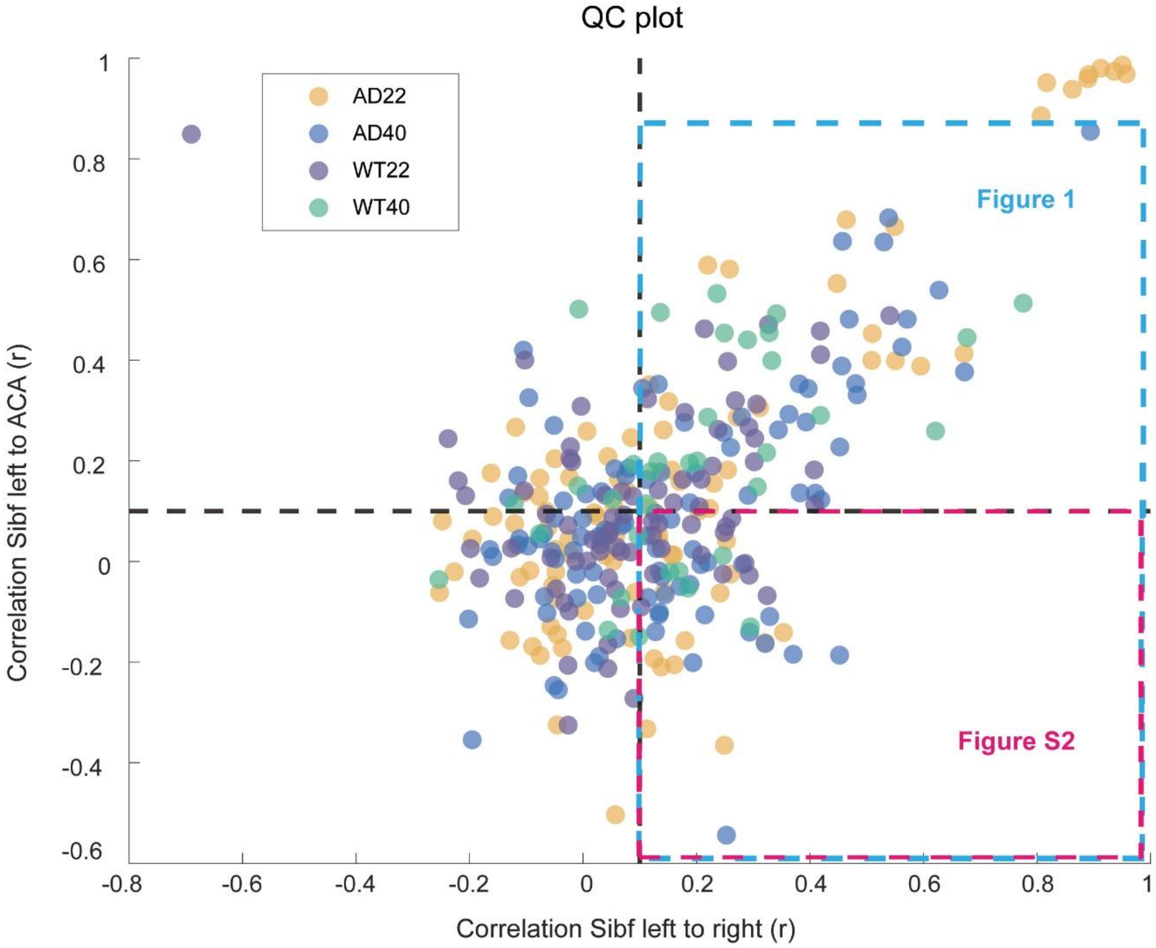

Figure S1. Quality control metric calculation for functional connectivity. Each dot represents the Pearson correlation coefficient of one epoch (96 repetitions) from one mouse. The x-axis is correlation between primary somatosensory cortex barrel field (Sibf) from left and right hemispheres. The y-axis is correlation between Sibf from the left hemisphere and the anterior cingulate area (ACA). Each mouse has 5 epochs. The horizontal and vertical black dashed lines are drawn at the 0.1 threshold for correlation. The main study used data only to the right of the vertical dashed line, Fig. S2 (below) used data only to the right of the vertical dashed line and below the horizontal dashed line. Data was also removed due to the threshold of three standard deviations (see Methods). Thus, the data in the blue dashed-line box is the data used in the main study, the data in the red dashed-line box is the data used in Fig. S2, below.

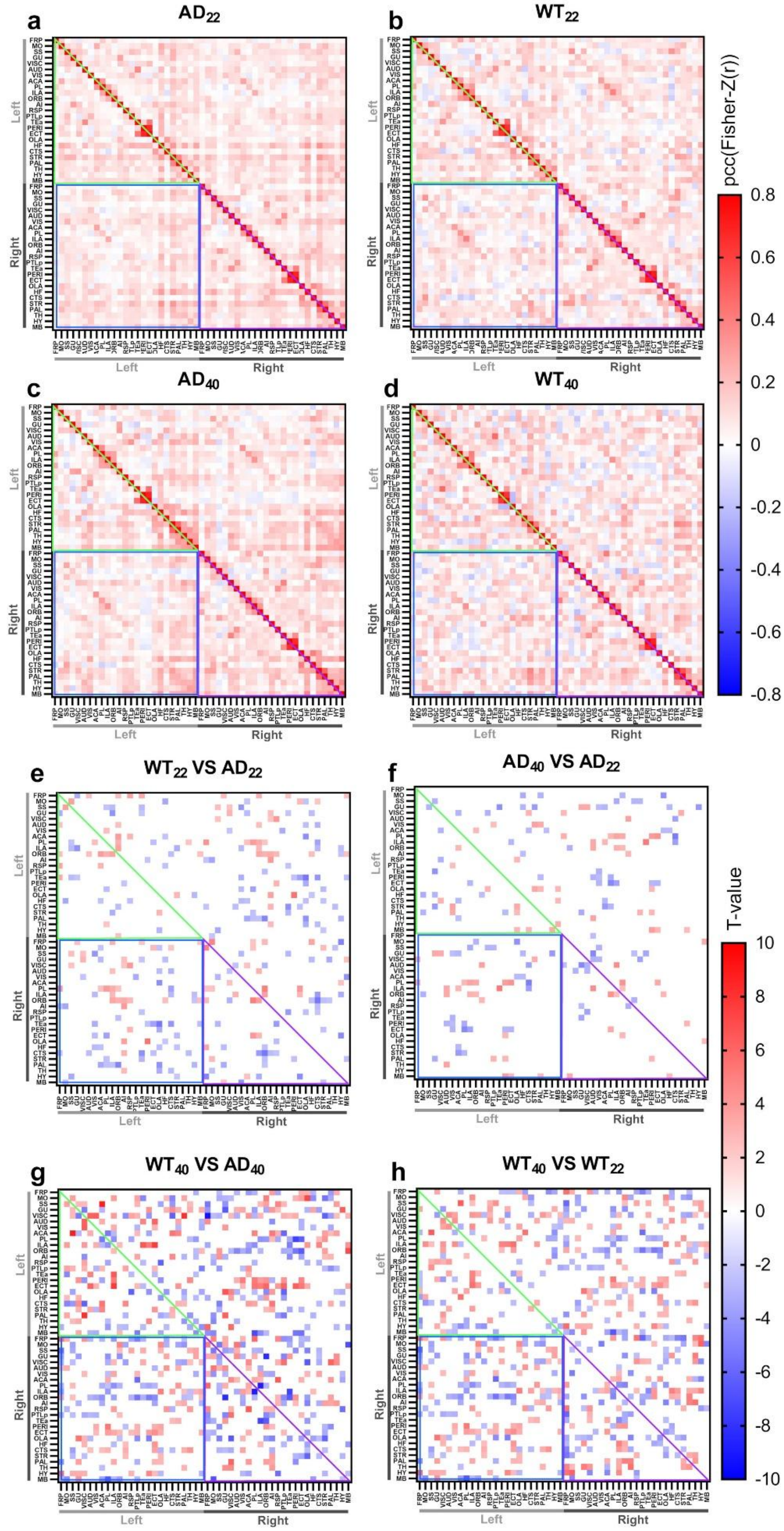
a
AD22
b
WT22
c
AD40
d
WT40
pcc(Fisher-Z(r))
0.8
0.6
0.4
0.2
0
-0.2
-0.4
-0.6
-0.8
e
WT22 VS AD22
f
AD40 VS AD22
g
WT40 VS AD40
h
WT40 VS WT22
T-value
10
8
6
4
2
0
-2
-4
-6
-8
-10
Left
Right
FRP
MO
SS
GU
VISC
AUD
VIS
ACA
PL
ILA
ORB
AI
RSP
PTLp
TEa
PERI
ECT
OLA
HF
CTS
STR
PAL
TH
HY
MB

Figure S2. Identical to Fig. 1 in the main text, except only functional connectivity values were kept that were both to the right of the horizontal dashed line and below the vertical dashed line shown in Fig. S1. All statistical tests and multiple comparison corrections were re-calculated for this figure.

Supplementary Tables

Abbreviation	Name	Allen number
FRP	Frontal pole	6–11
MO	Somatomotor areas	12–29
SS	Somatosensory areas	30–106
GU	Gustatory areas	107–113
VISC	Visceral areas	114–120
AUD	Auditory areas	121–156
VIS	Visual areas	157–219
ACA	Anterior cingulate area	220–237
PL	Prelimbic area	238–244
ILA	Infralimbic area	245–251
ORB	Orbital area	252–277
AI	Agranular insular area	278–296
RSP	Retrosplenial area	297–338
PTLp	Posterior parietal association areas	339–359
TEa	Temporal association areas	360–366
PERI	Perirhinal area	367–372
ECT	Ectorhinal area	373–378
OLA	Olfactory areas	379–453
HF	Hippocampal formation	454–554
CTXsp	Cortical subplate	555–570
STR	Striatum	571–607
PAL	Pallidum	608–638
TH	Thalamus	641–714
HY	Hypothalamus	715–805
MB	Midbrain	806–881

Table S1. Abbreviation, full name, and numbers from the Allen mouse brain atlas for brain regions used in our study.

		AD_{22}-L	AD_{22}-R	WT_{22}-L	WT_{22}-R	AD_{40}-L	AD_{40}-R	WT_{40}-L	WT_{40}-R
**FRP**	r	0.4703	0.6716	0.79	0.7548	0.7229	0.8807	0.7691	0.7226
	p	0.0897	0.0085*	0.0008*	0.0018*	0.0035*	<0.0001*	0.0013*	0.0035*
**GU**	r	0.818	0.8508	0.8624	0.8409	0.8009	0.7984	0.5745	0.672
	p	0.0004*	0.0001*	<0.0001*	0.0002*	0.0006*	0.0006*	0.0316	0.0085*
**ORB**	r	0.1463	0.02843	0.3874	0.5075	0.1461	0.1395	0.3537	0.2632
	p	0.6177	0.9232	0.1711	0.064	0.6182	0.6343	0.2147	0.3632
**AI**	r	0.2323	0.5476	0.5437	0.3086	0.2296	0.3038	0.04934	0.1615
	p	0.4241	0.0427	0.0444	0.2831	0.4298	0.2909	0.867	0.5812
**TEa**	r	0.2545	0.4123	0.6069	0.4017	0.518	0.5562	0.3172	0.4149
	p	0.3799	0.1429	0.0214	0.1545	0.0578	0.0389	0.2691	0.1402
**PAL**	r	0.6526	0.6805	0.8408	0.8109	0.7893	0.6578	0.7803	0.7478
	p	0.0114*	0.0074*	0.0002*	0.0004*	0.0008*	0.0106*	0.001*	0.0021*
**HF**	r	0.5062	0.5949	0.4097	0.4615	0.4884	0.4634	0.507	0.4524
	p	0.0648	0.0248	0.1458	0.0967	0.0764	0.0951	0.0643	0.1043
**TH**	r	0.7239	0.6877	0.7069	0.6142	0.8148	0.7683	0.8041	0.7651
	p	0.0034*	0.0066*	0.0047*	0.0194	0.0004*	0.0013*	0.0005*	0.0014*
**HY**	r	0.6827	0.6405	0.8591	0.8156	0.8108	0.8062	0.7667	0.7352
	p	0.0071*	0.0136*	<0.0001*	0.0004*	0.0004*	0.0005*	0.0014*	0.0027*

Table S2. The r and *p*-value of the correlation between FC and SC in different brain regions. * represents correlation significance after SgoF test.

	22 weeks AD	22 weeks WT	40 weeks AD	40 weeks WT
FRP	-0.83	-0.42	-0.26	-0.38
GU	0.40	0.017	-0.15	-0.40
ILA	-1.21	-0.57	-0.53	-0.86
AI	-0.69	-0.44	-0.30	-0.81

Table S3. Mean slope of the linear regression between FC and FA of different groups. The difference in slope between different groups was not significant ($p > 0.05$). See also Fig. 4.
